# Supplementary material for: Prolyl Endopeptidase-Like Facilitates the α-Synuclein Aggregation Seeding, and This Effect Is Reverted by Serine Peptidase Inhibitor PMSF
Source: Biomolecules. 2020 Jun 25;10(6):962. doi: 10.3390/biom10060962 (PMC7355856; doi:10.3390/biom10060962)
Supplement: Supplementary file 1 [file biomolecules-10-00962-s001.pdf]

Supplementary Materials

Figure S1. Multiple sequence alignment with predicted local (secondary) structures.

```
Human_PREPL      1   MQQKTKLFLQALKYSIPHLGKCMQKQHLNHYNFADHCYNRIKLKKYHLT-    49
Human_POP        1   ML----------------------------SFQYPDVYRDETAVQDYHG-    21
OPB_TB           1   -----------------------------MQTERGPIAAHRPHEVVFGKV    21
Consensus_ss:                                                  eeeee

Human_PREPL     50   -------------KCLQNKPKISELARNIPSRSFSCKDLQPVKQENEKPL    86
Human_POP       22   -------------HKICDPYAWLEDPD-------SEQTKAFVEAQNKITV    51
OPB_TB          22   EGEDRGANPMDPPRRKVDPLFWLRDDN-----RADPEVLAHLHLEKDYYE    66
Consensus_ss:                     eeee   hhh            hhhhhhhhhhhhhhh

Human_PREPL     87   PENM---DAFEKVRTKLETQPQEEYEIINVEVKHGGFVYYQEGC---CLV   130
Human_POP       52   PFLEQC-PIRGLYKERMTELYDYPK--YSCHFKKGKRYFYFYNTGLQNQR    98
OPB_TB          67   KRAVDIKDLAETIYQEHISHIEETD--MSAPYVYDRFLYYTRDVKGLSYK   114
Consensus_ss:        hhhh   hhhhhhhhhhhhhh          eee  eeeeeee     ee

Human_PREPL    131   RSKDEEAD-----NDNYEVLFNLEELKLDQP---FIDCIRVAPDEKYVAA   172
Human_POP       99   VLYVQDSL-----EGEARVFLDPNILSDDGTV--ALRGYAFSEDGEYFAY   141
OPB_TB         115   LHCRVPAGKTPGEGEDEEIVLDENKLAEGKSFCVVGCVAPAPPEHALVAY   164
Consensus_ss:        eeeeee           eeee hhh     ee  eeeeeee     eeee

Human_PREPL    173   KIRTEDSEASTCVIIKLSDQPVMEASFPNVSSFEWVKDEEDEDVLFYTFQ   222
Human_POP      142   GLSASGSDWVTIKFMKVDGAKELPDVLERVKFSCMAWTHDGKGMFYNSYP   191
OPB_TB         165   SVDYCGDEVYSIRFVRDVVADKVEGT-----NGSVVWGPNAECFFYITKD   209
Consensus_ss:        ee      eeeeeeee     eeee       eeeee     eeeeeee

Human_PREPL    223   R-----------NLRCHDVYRATFGDNKR-NERFYTEKD-PSYFVFLYLT   259
Human_POP      192   QQDGKSDGTETSTNLHQKLYYHVLGTDQSEDILCAEFPDEPKWMGGAELS   241
OPB_TB         210   A-----------SKRDNKVWRHIIGQPQSEDVCLYTDDD-PLFSVGVGKS   247
Consensus_ss:                        eeeeeee        eeee      eeeeeeee

Human_PREPL    260   KDSRFLTINIMNKTTSE--VWLIDGLSPWDPPVLIQKRIHG-----V-LY   301
Human_POP      242   DDGRYVLLSIREGCDPVNRLWYCDLQQESSGIAGILKWVKLIDNFEGEYD   291
OPB_TB         248   GDGKTLIICSMSSETSESLLLDLRKGVKHNTLEMVRPREKG-----VRYT   292
Consensus_ss:            eeeeee      eeeeeeee       eeee             ee

Human_PREPL    302   YVEHRDDELYILTNVGEPTEFKLMRTAADTPAIMNWDLFFTMKRNTKVID   351
Human_POP      292   YVTNEGAVFTFKTNRQ-SPNYRVINIDFRDPEESKWKVLVPEHEKDVLEW   340
OPB_TB         293   VEMHGTDTLIVLTNKDKCVNGKVVLTKRSAPT-DWGTVLIPHDDKVTIDD   341
Consensus_ss:        eee    eeeeee       eeeeeee        eeeee     eeeee

Human_PREPL    352   LDMFK-DHCVLFLKHSNLLYVNVIGLAD------DSVRSLKLPPW----A   390
Human_POP      341   IACVRSNFLVLCYLHDVKNILQLHDLTT------GALLKTFPLDV----G   380
OPB_TB         342   VAVFA-KFAVLSGRRDGLTRVWTVRLGPDSLFSSATLKELHFDEPVFTAH   390
Consensus_ss:        eeee   eeeeeee   eeeeeeee           eeeee

Human_PREPL    391   CGFIMDTNSDPKNCPFQLCSPIRPPKYYTYKFAEGKLFEETGHED----P   436
Human_POP      381   SIVGYSGQKKDTEIFYQFTSFLSPGIIYHCDLTKEELEPRVFREVTVKGI   430
OPB_TB         391   VVCSQMKTYDASLLRLRYSSMTTPTVWYDEDVLSGERKVVKARKVG-GGF   439
Consensus_ss:        eeeeee      eeeeee       eeeeee     eeeeee

Human_PREPL    437   ITKTSRVLRLEAKSKDGKLVPMTVFHKTDSEDLQKKPLLVHVYGAYGMDL   486
Human_POP      431   DASDYQTVQIFYPSKDGTKIPMFIVHKKSIKLDGSHPAFLYGYGGFNISI   480
OPB_TB         440   QSKNYVCRRELATAPDGTKVPISLVYDTSIDLKKPNPTMLYGYGSYGICI   489
Consensus_ss:              eeeeeee     eeeeeeee          eeeeee

Human_PREPL    487   KMNFRPERRVLV-DDGWILAYCHVRGGGELGLQWHAD-GRLTKKLNGLAD   534
Human_POP      481   TPNYSVSRLIFVRHMGGILAVANIRGGGEYGETWHKG-GILANKQNCFDD   529
OPB_TB         490   EPEFNSRFLPYV-DRGMIYAIAHVRGGGEMGRTWYEVGGKYLTKRNTFMD   538
Consensus_ss:             hhhhhhh h   eeeee         hhhhhh hhhh    hhhh
                                                *
Human_PREPL    535   LEACIKTLHGQGFSQPSLTTLTAFSAGGVLAGALCNSNPELVRAVTLEAP   584
Human_POP      530   FQCAAEYLIKEGYTSPKRLTINGGSNGGLLVAACANQRPDLFGCVIAQVG   579
OPB_TB         539   FIACAEHLISSGLTTPAQLSCEGRSAGGLLVGAVLNMRPDLFHVALAGVP   588
```

```
Consensus_ss:       hhhhhhhhhh     hhheeeeee hhhhhhhhhhh     eeeeeee
                                  S1
Human_PREPL    585  FLDVLNTMMDTTLPLTLEELEEWGNPSSDEKHKNYIKRYCPYQNIKPQ--  632
Human_POP      580  VMDMLKFHKYT---IGHAWTTDYGCS-DSKQHFEWLVKYSPLHNVKLPEA  625
OPB_TB         589  FVDVMTTMCDPSIPLTTGEWEEWGNP-NEYKFFDYMNSYSPIDNVRAQ--  635
Consensus_ss:          hhhh         hhhhhhh      hhhhhhhhh  hhhhhh
                                   *
Human_PREPL    633  ---HYPSIHITAYENDERVPLKGIVSYTEKLKEAIAEHAKDTGEGYQTPN  679
Human_POP      626  DDIQYPSMLLLTADHDDRVVPLHSLKFIATLQYIVGRSRKQ------SNP  669
OPB_TB         636  ---DYPHLMIQAGLHDPRVAYWEPAKWASKLRELKT----D------SNE  672
Consensus_ss:              eeeeee      hhhhhhhhhhhhh                    e
                              *
Human_PREPL    680  IILDIQPGGNHV----IEDSHKKITAQIKFLYEELGLDSTSVFEDLKKYL  725
Human_POP      670  LLIHVDTKAGHGAGKPTAKVIEEVSDMFAFIARCLNVDWIP---------  710
OPB_TB         673  VLLKMDLESGHFSASDRYKYLRENAIQQAFVLKHLNVRQLL-----RK--  715
Consensus_ss:       eeeeee          hhhhhhhhhhhhhhhhhh

Human_PREPL    726  KF  727
Human_POP           --
OPB_TB              --
Consensus_ss:
```

Figure S1. Multiple sequence alignment with predicted local (secondary) structures. Sequences of human PREPL (longer isoform), human POP and oligopeptidase B from *T. brucei* (OPB_TB) were aligned at the PROMALS3D multiple sequence and structure alignment server (*PROMALS3D: a tool for multiple sequence and structure alignment. Jimin Pei, Bong-Hyun Kim and Nick V. Grishin. Nucleic Acids Res. 2008 36(7):2295-2300*). The α-helix sections are shown in red letters and the β-strands in blue letters. For PREPL these secondary structural elements were predicted by the PROMALS3D whereas for POP they were based on the PDB 3EQ7 and for OPB_TB on the PDB 4BP8. Asterisks are placed to show the residues of the conserved catalytic triad, which are also highlighted in gray. The region in which there are residues important to the substrate binding S_1 sub-site of POP and OPB_TB and consequently thought to form also the S_1 sub-site of PREPL is boxed.

Comments on Figure S1. It is noteworthy that using only regular default sequence alignments (such as ClustalW), the histidine residue of the catalytic triad was incorrectly aligned: The PREPL His696 was incorrectly aligned with the His680 of POP and His683 of OPB_TB, while, the correct residue of the catalytic triad of PREPL is the His690, as already experimentally verified by site-directed mutagenesis (*Jaeken, J.; Martens, K.; François, I.; Eyskens, F.; Lecointre, C.; Derua, R.; Meulemans, S.; Slootstra, J.W.; Waelkens, E.; De Zegher, F.; et al. Deletion of PREPL, a gene encoding a putative serine oligopeptidase, in patients with hypotonia-cystinuria syndrome. Am. J. Hum. Genet. 2006, 78, 38–51.*). However, by making the alignment by PROMALS, considering the secondary structures, as showed above, the PREPL His690 is correctly aligned.

Figure S2. Comparison of the CD spectra obtained for PREPL and POP.

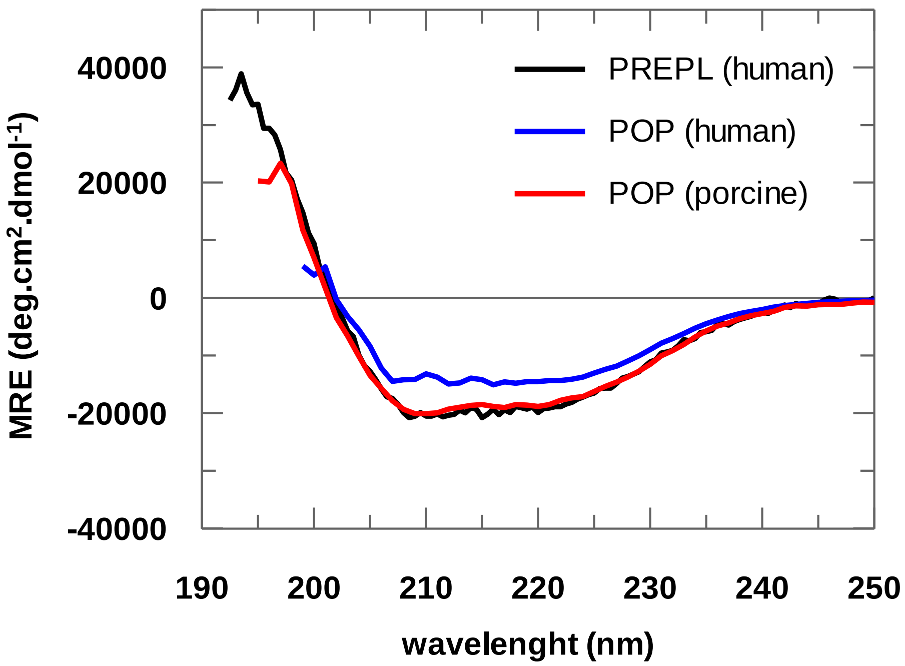

Figure S2. Comparison of the CD spectra obtained for *human* PREPL, *human* and *porcine* POP.

Comments on Figure S2. The spectrum obtained for human PREPL is more intense than the spectrum obtained for human POP. This most probably arise from the experimental errors intrinsic of the protein concentration measurements. However, in Figure 1S, it can be observed two helix sections predicted to exist in the PREPL structure that is not present in the structure of POP (one near the PREPL N-terminus, from residue number 3 to 16 or even to residue number 22, and another near the C-terminus, from residue number 715 to residue 725). These two extra helix sections may indicate that PREPL has a higher α-helix content than POP. Therefore, in order to check such difference a parallel CD measurement with the porcine POP (which we have already prepared at our lab) was done. The CD spectra obtained for human PREPL and porcine POP superimposed perfectly. Confirming that the difference in the intensities of the CD spectra observed between the human PREPL and human POP probably is due to the errors intrinsic to our practical methodologies. An additional indication that this difference relies on practical manipulations reasons is that: we were able to use a 0.2 mm cuvette for the measurements with human PREPL and porcine POP what permitted the collection of reliable spectra (HT < 600V) at wavelengths as low as ~192 nm. However, we had to work with a 1 mm cuvette for samples of human POP to decrease the signal to noise ratio, but this larger pathlength increased the total absorbance of light and then we could just get a reliable CD signal until 199 nm.

Figure S3. Adjusted sensorgrams obtained in the SPR assays with PREPL or POP immobilized and α-Syn as the analyte injected at increasing concentrations.

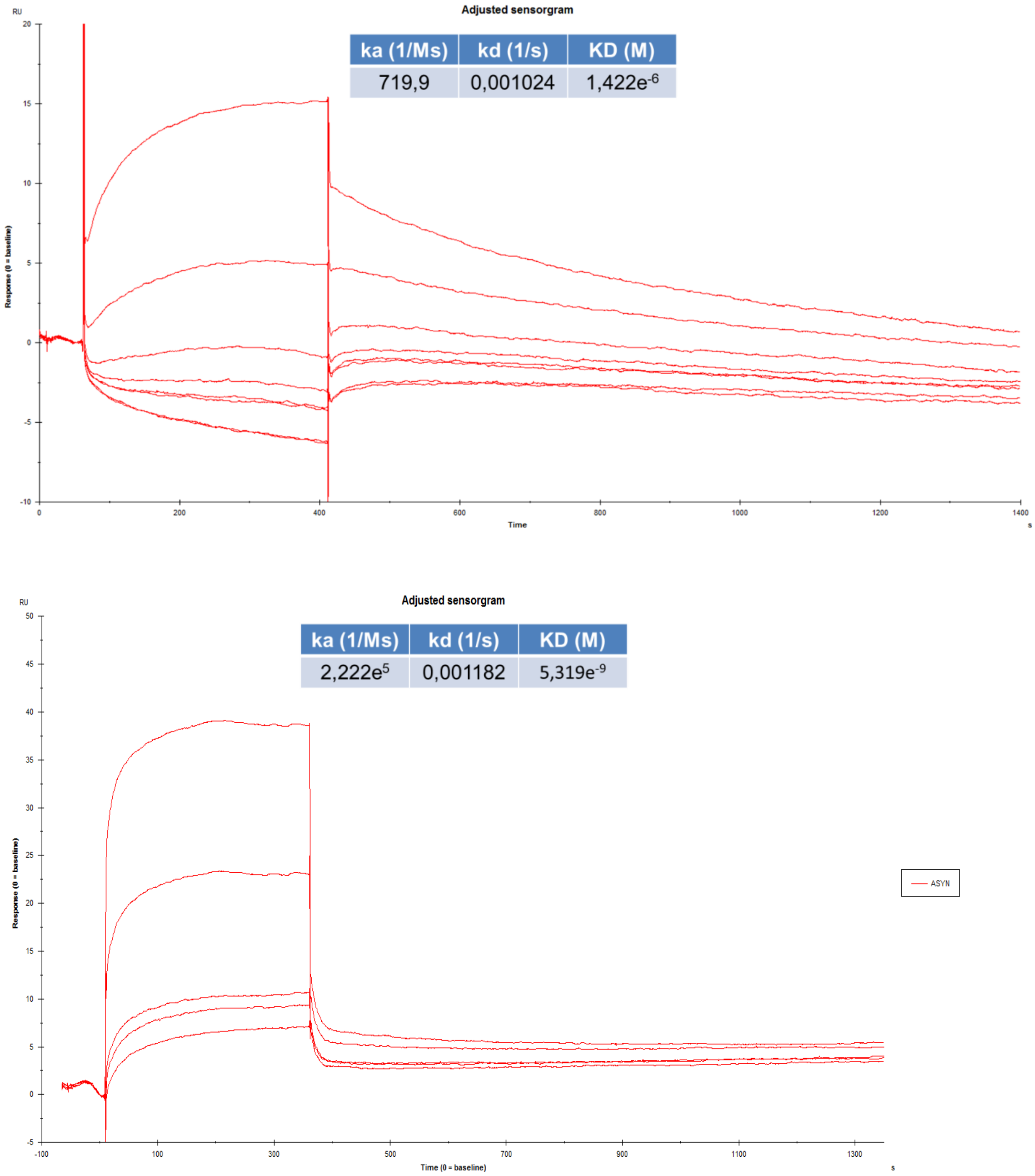

Figure S3. Adjusted sensorgrams obtained in the SPR assays with PREPL (upper panel) or POP (lower panel) immobilized and α-Syn as the analyte injected at increasing concentrations.

Comments on Figure S3. Although we could not observe the saturation of the dose-response curve of the titrations with α-Syn of the POP immobilized on the sensor chip, by the association constants obtained in the SPR analysis (ka values shown in the Figure S3), it can be affirmed that the affinity of the α-Syn for POP is higher than its affinity by PREPL, mainly because of a higher association constant.
